# Doctor, Bot, or Both: Questioning the Medicolegal Liability of Artificial Intelligence in Indian Healthcare

**DOI:** 10.7759/cureus.69230

**Published:** 2024-09-11

**Authors:** Satvik N Pai, Madhan Jeyaraman, Naveen Jeyaraman, Sankalp Yadav

**Affiliations:** 1 Orthopedics, PES University Institute of Medical Sciences and Research, Bengaluru, IND; 2 Orthopedics, South Texas Orthopedic Research Institute, Texas, USA; 3 Clinical Research, Virginia Tech India, Dr. M.G.R Educational and Research Institute, Chennai, IND; 4 Orthopedics, ACS Medical College and Hospital, Dr. M.G.R Educational and Research Institute, Chennai, IND; 5 Medicine, Shri Madan Lal Khurana Chest Clinic, New Delhi, IND

**Keywords:** artificial intelligence, healthcare, indian healthcare, liability, medicolegal

## Abstract

Artificial intelligence (AI) is rapidly transforming the landscape of healthcare, with its applications ranging from diagnostics to robotic surgery. As India swiftly integrates AI into its healthcare system, a critical question arises: who bears the liability when AI-driven medical decisions go wrong? This article explores the complexities of assigning legal responsibility for AI in healthcare, particularly within the Indian context. While India's legal framework, including the Information Technology Act, intersects with AI usage, it lacks specific provisions addressing AI liability. The National Strategy for AI, although a step forward, does not resolve the ambiguities surrounding accountability. The article also examines how other countries, particularly the European Union and the United States, are beginning to tackle these challenges through proposed directives and regulations. However, the absence of clear, comprehensive laws worldwide reflects the inherent difficulties in legislating AI stemming from its autonomous nature, the "black box" problem, and the multifaceted roles of stakeholders. Until robust legal frameworks are established, the article cautions healthcare professionals to exercise vigilance when using AI tools, recognizing that they may still bear significant liability. This evolving legal landscape underscores the need for continued deliberations in AI regulations.

## Editorial

Artificial intelligence, or AI as it is called, is the new "in thing." In the span of just a couple of years, its application has expanded into almost all fields. Healthcare is no different, with several new start-ups focusing specifically on the application of AI and existing service providers being quick to adapt to the changing landscape. You may like it or dread it, but it is inevitable that AI will play a role in the future of healthcare across the world. We are already seeing the incorporation of AI in healthcare in various specialties of medicine. The question of what the role of AI will be in healthcare in India seems to have an answer that is evolving every day. India has already been quick to adopt AI in healthcare, from diagnosis to data analysis and robotics. But there is one question that yet remains to be answered: When the bot goes bust, who is to blame?

Legal liability in India

There is no specific law in India regarding AI yet. However, there are certain legal frameworks that would intersect with the use of AI and can be applicable to various aspects of AI. The predominant one is the Information Technology Act. While it came into effect in the year 2000, much before AI became what it is today, it does deal with cyber security, data protection, and electronic records, which can be indirectly applied to AI. In order to provide some guidance on the Indian government's stance on AI, the NITI Aayog released the National Strategy for AI in 2018; however, it serves more as a roadmap for AI development in India than a regulatory or legal document [1]. While it highlights the need for fairness, transparency, accountability, and the prevention of bias in AI, it does not provide concrete guidelines or proposals on how liability should be managed. It, however, does acknowledge the complexity of the issue of liability, recognizing that AI systems challenge traditional notions of liability due to their autonomous decision-making capabilities [2]. Considering this, it is not surprising that there is still no clarity when it comes to liability for AI in healthcare. So if AI is relied upon to treat a patient and something goes wrong, we still don’t know who is going to be held liable in India. But do other countries have an answer to this?

Looking at liability in other countries around the world

Most nations, like India, are still in the process of refining their legal frameworks to accommodate the unique challenges AI presents. Leading the way is the European Union, with its proposed AI liability directive, which seeks to make it easier for victims to claim compensation when AI systems cause harm [3]. However, even this directive is still being negotiated and has not yet been enforced. The European Union is also updating its product liability directive to clarify that AI and digital products fall under product liability rules, meaning that manufacturers can be held liable for damages caused by defective AI systems. No individual nation has yet come out with a comprehensive AI law. In the United States of America, the US Food and Drug Administration (FDA) regulates AI-based medical devices, ensuring that they meet safety and effectiveness standards. The US FDA has cleared or approved several AI-driven healthcare products, such as the IDx-DR system, the first AI system approved for autonomously diagnosing diabetic retinopathy [4]. If such an AI system causes harm, liability could arise under existing product liability laws. We are only now beginning to see litigations in courts of law relating to the liability for AI, and once verdicts are passed in such cases, they are likely to become very useful case laws that will help determine the legal liability more accurately.

Why is it so complicated?

At first glance, it may seem like the liability is straight-forward. If an AI system falters, and that leads to damage to a patient, the manufacturer of the software or medical device should be held liable. But a closer look reveals the various levels of this liability. AI systems do not claim to be 100% accurate, and even if this is disclosed to the user beforehand, the question of liability remains when the system is inaccurate or wrong The technical aspect that makes forming any blanket law on the liability of AI so difficult is the complexity and varied autonomy of AI systems. There are AI systems that do not act with complete autonomy and function more like tools for the use of a medical professional. They are more like guides to a medical professional. For such AI systems, the medical professional acts as the final decision-maker and hence will shoulder some of the liability [5]. While the medical professional would be required to use their own discretion in such situations, they can argue that the information or guidance provided by the AI was inaccurate, thereby making the process of making the right decision difficult for the user. This "black box" nature of AI, where even the user may not know the process, source, or algorithm for its outputs, makes it challenging to establish causality and accountability in legal terms. Then there are those AI systems that can operate autonomously, making decisions without direct human intervention. When such an AI system malfunctions or misses a diagnosis, causing harm to the patient, who then becomes responsible? The developers of the AI system or the medical professional/institution that deployed such an AI system. These are all the questions that may not have a straightforward, simple answer. It is a nuanced subject that will probably need a couple more years, some case laws, and a better understanding of AI and its applicability in healthcare to really shed more light on it.

What do we do till then?

Considering the ambiguity around liability for AI use in healthcare, we must be cautious in our approach. One must realize that for current AI systems, the majority of which are not fully autonomous, a large part of the liability will still be with the medical professionals or institutions using the AI. Most of the applications of AI in the healthcare sector are still seen as assistive, where the AI is a tool that a medical professional can use to improve various aspects of healthcare, but a tool at the end of the day. When things go wayward, it is likely the individual using that tool is still going to have to answer a considerable number of questions. That being said, as AI evolves, so will the legislature related to it, and we are likely to have more clear guidelines and laws in relation to it. Until then, we must choose our tools with caution and be wary of handing over the reins to AI just yet.
